# Patient coaching for deep inspiration breath hold decreases set-up duration and left anterior descending artery dose for left-sided breast cancer radiotherapy

**DOI:** 10.1007/s00520-025-09446-1

**Published:** 2025-04-16

**Authors:** Aysegul Ucuncu Kefeli, Umut Diremsizoglu, Sevda Erdogan, Aysegul Unal Karabey, Aykut Oguz Konuk, Berna Tirpanci, Maksut Gorkem Aksu, Emine Binnaz Sarper

**Affiliations:** Department of Radiation Oncology, Kocaeli University School of Medicine, Kabaoglu Mahallesi, Baki Komsuoglu bulvarı No:515, Umuttepe, 41001 İzmit Turkey

**Keywords:** Deep inspiration breath-hold, Left-sided breast cancer, Real-time position management

## Abstract

**Purpose:**

The purpose is to show the impact of patient coaching and home practice using the deep inspiration breath hold (DIBH) technique on radiation treatment set-up times and cardiac at-risk doses.

**Methods:**

The study involved patients who received tangential field radiotherapy using the DIBH technique for treating left breast cancer. Patients were divided into two groups: the first group consisted of those who received coaching from an oncology nurse and were given an instruction sheet at least 1 week before the computed tomography (CT) simulation. The second group consisted of those who were only taught how to hold their breath by the radiation technician on the simulation day and without further education. During treatment, the patients were monitored using the Varian RPM™ respiratory gating system, and 2D kV orthogonal imaging was performed daily. The setup duration of each patient was noted and compared between treatment groups. For each patient, the dose-volume histograms (DVHs) of the heart, LAD (left anterior descending artery), were calculated and compared for both coached DIBH (cDIBH) and non-coached DIBH (ncDIBH).

**Results:**

Thirty-six coached and 28 non-coached patients were identified. Compared with ncDIBH, coached patients were older (55.5 versus 46.5, *p* = 0.003) and had a significantly higher BMI (body mass index) (29.95 versus 26.32 kg/m^2^, *p* = 0.006). Nevertheless, in more than half of the treatment fractions, the set-up duration was detected to be statistically longer in the ncDIBH group than in the cDIBH group. Additionally, the LAD max dose was significantly lower in the cDIBH group (36.5 versus 29.5, *p* = 0.02).

**Conclusion:**

Coaching at least 1 week before the simulation with an instruction sheet decreased the set-up duration, and the cardiac LAD max dose should be further decreased by this method.

## Introductıon

With an estimated 2.3 million new cases per year and accounting for 11.6% of all cancers worldwide, breast cancer is the most commonly diagnosed cancer in women, according to Globocan 2022 data [1]. The role of radiotherapy in the treatment of breast cancer plays an essential role since it has been shown to significantly reduce the risk of local recurrence and improve overall survival. However, investigations have also shown that decreasing mortality rates indicate a potential patient population at higher risk of cardiac injury. Many researchers have assessed long-term cardiac toxicity and mortality from left-sided breast and chest wall radiation [2–4]. An increased risk of major coronary events is linked to increases in the mean cardiac dose and the dose to cardiac substructures. Darby et al. demonstrated a linear increase in the risk of major coronary events induced by radiation with the mean heart dose (MHD) increasing by 7.4% per gray, without any observable threshold dose [5].

The sparing of cardiac dose through deep inspiration breath hold (DIBH) has been extensively researched. DIBH techniques decrease the dose to the heart and coronary arteries significantly compared with free breathing (FB) [6, 7]. In this technique, each radiation treatment is administered during an actively participated controlled deep breath hold. Patients should take deep inspiration and hold for approximately 20 s during the simulation and treatment. In most patients, during deep inspiration, the heart moves to the right and inferiorly due to the expansion of the chest cavity and lung volume. This displacement of the heart caused the target structures for radiation therapy (breast, chest wall) to move farther away from the heart. When the larger distance between the heart and target structures is combined with rigorous 3D conformal or intensity-modulated imaging-based radiation dose planning, the heart dose can be reduced while maintaining acceptable coverage to the breast or chest wall target structures.

While DIBH has proven to be an effective technique for reducing cardiac exposure in patients with left-sided breast cancer, previous studies primarily focused on minimizing radiation-induced cardiac toxicity by lowering doses to the heart [7]. Since then, many studies have shown similar results, designed in the same way [8, 9]. However, less attention has been given to the effective implementation of DIBH, which relies on patient training and cooperation. There are audio and visual coaching systems used to improve the reproducibility of DIBH, but there are still challenges in the clinical implementation of DIBH [10]. DIBH is a complex technique that must be reproducible daily (5 days a week). In actual clinical practice, success rates vary depending on the degree of patient cooperation [11]. Previous studies have indicated that patient coaching may improve the success rate of DIBH [12]. A sustained and steady DIBH is essential to maintain the necessary distance between the heart and the radiation beam target. Thus, the patient’s precise cooperation is crucial to the effectiveness of each daily DIBH.

Even though our clinic has been performing this technique since 2018, the radiation technician has always told patients to hold their breath under deep inspiration either 1 or 2 days before the computed tomography (CT) simulation or two times on the day of the procedure. Usually, because of cooperation issues, not every patient was able to complete the CT scan. Among the patients who underwent successful CT simulation, most experienced cooperation problems on the treatment day and could not perform DIBH optimally, leading to increased treatment set-up times. This treatment also increases patient anxiety and workload. To address this problem, in 2023, we began providing coaching to patients 1 week before the procedure, and our radiation oncology nurse also conducted teaching, as per a page of instructions that the radiation oncologist and nurse had created. Patients will receive this page at least 5 days before the simulation for use at home.

This study aims to evaluate the effect of patient coaching before treatment by an educated nurse and home training on DIBH setup time and cardiac dose.

## Methods and materials

### Patient selection and factors

The study included patients who underwent tangential field radiotherapy using the DIBH technique for the treatment of left breast cancer at the Kocaeli University Radiation Oncology Clinic between January 2020 and April 2024. This retrospective study was approved by the Institutional Review Board. Patients were divided into two groups: the first group that received coaching from a nurse in addition to receiving the instruction sheet; the second group consisted of those who were only taught how to hold their breath by the radiation technician without receiving any further education. The exclusion criteria were patients who received internal mammary chain irradiation. A total of 64 female patients were identified retrospectively. Thirty-six of them were in the coached group and 28 were in the non-coached group. The study evaluated patient factors that could affect the ability to perform DIBH, including age, body mass index (BMI), education level, disease stage, history of cardiopulmonary diseases (such as asthma, chronic obstructive pulmonary disease, and heart failure), and use of adjuvant and/or neoadjuvant chemotherapy.

### Coaching

**Fig. 1:**
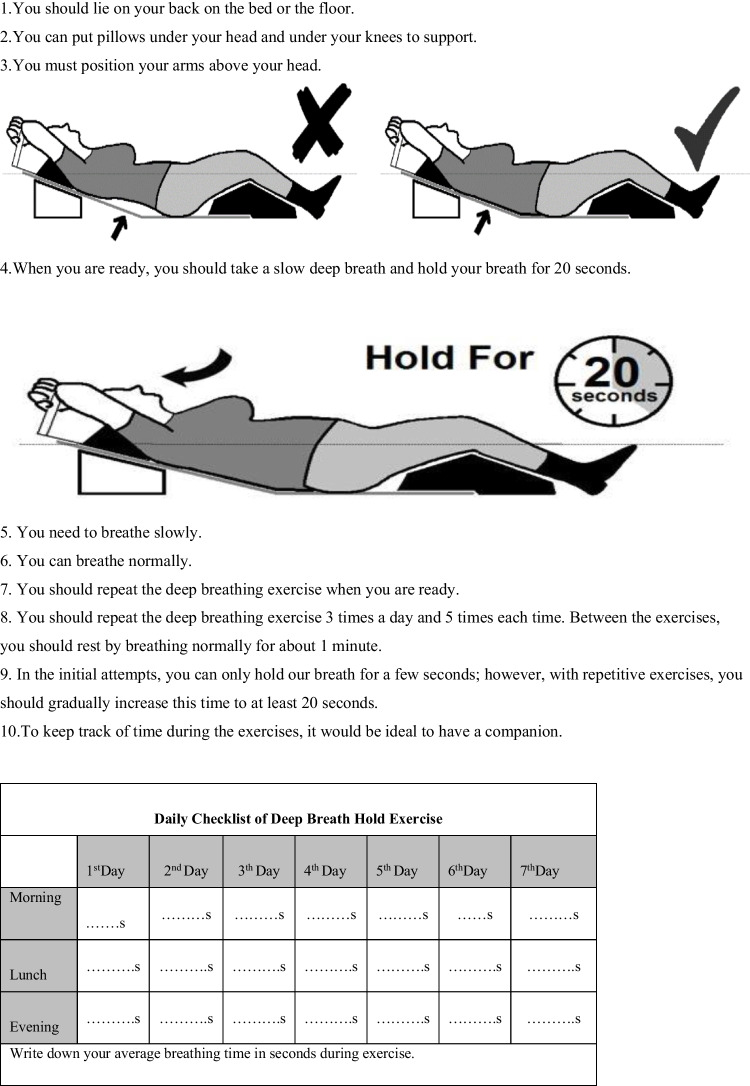
Instructions for deep ınspiration breath hold

Coaching sessions were conducted after the radiotherapy visit by our educated oncology nurse and lasted approximately 10 to 15 min. Patients were told to follow home instructions at least 1 week before the simulation. They should lie in the treatment position with arms above their head; take a slow, deep breath; and hold their breath for 20 s. Then, breathe slowly and normally and repeat the deep breathing exercise three times a day and five times each time. Between the exercises, the patient should rest by breathing normally for approximately 1 min. In the first trial, the participants held their breath for shorter seconds. In the repetitive exercises, they should increase the time and continue the exercises for at least 20 s. They were also advised to fill out a checklist for their daily exercise. The instruction sheet is shown in Fig. 1. During the simulation, the physician confirmed that the patient practiced at home.

The non-coached DIBH (ncDIBH) group received instructions from the radiation technician according to the routine protocol on the day of the simulation.

### The DIBH method and simulation

The voluntary DIBH technique was used during the CT simulation. Patients were immobilized supine via a breast board with their left arm up. The Varian RPM™ Respiratory Gating System (Varian Medical Systems, Palo Alto, CA), version 1.7.5, was used for respiratory control. The RPM fiducial box was positioned on the patient’s upper abdomen midway between the xiphoid process and the umbilicus. Patients were asked to breathe freely and then inhale and hold their breath comfortably for at least 20 s and were tracked throughout the scan duration using the RPM. The inhale level to reach for all coached and noncoached patients was not “too deep” but “moderately deep” that we defined as roughly 70 to 80% of the maximum BH of each individual patient. The gating window was set to 5 mm, and this level was utilized to perform DIBH movements during the treatment phases.

### Treatment planning and organs at risk

The planning system was Eclipse 13.6 (Varian Medical Systems, Palo Alto, CA, USA). A 3D conformal radiotherapy (3D-CRT) or intensity-modulated radiotherapy (IMRT) plan was generated using the tangential and field-in-field techniques. The prescribed doses of the treatment plans were 42.4–50 Gy in 16–25 fractions using 6 MV photons, with or without sequential boost to the tumor bed. All plans were generated with at least 95% isodose, encompassing the entire breast, and no hot spots exceeded 110% isodose.

Cardiac structures were contoured following the Feng cardiac atlas guidelines. For clinical target volume (CTV) delineation, RTOG Breast Atlas contours were used [13]. For patients treated with 42.4 Gy in 16 fractions (hypofractionation regimen), the dose to the heart and LAD was corrected to 2 Gy per fraction, α/β = 2. Dosimetric analysis for normal tissues included maximum heart dose, mean heart dose (MHD), volume receiving 5 Gy (HeartV5), volume receiving 20 Gy (HeartV20), and volume receiving 30 Gy (HeartV30), max LAD dose and mean LAD dose. All of the OAR doses were obtained from the treatment planning system.

### Patient set-up

RT was delivered using a Varian Trilogy™ Linac. All patients immobilized supine, by means of a breast board with left arm up. The isocenter is defined based on the reference skin marks placed on the patient’s skin during the CT scan. The setup and positioning were verified with two-dimensional (2D) kV orthogonal imaging, both anteroposterior and lateral which were subsequently taken daily. The setup duration was calculated retrospectively from the first kV orthogonal imaging time to the treatment start time, as recorded on the treatment planning computer. Each patient’s set-up preparation time [s] was noted and compared between treatment groups for 16 fractions. Because total treatment times might be biased due to the amount of MU required to deliver the dose to the patient, we only analyzed set-up duration of daily pre-treatment kV imaging.

## Statistical analysis

IBM SPSS Statistics for Windows, Version 21.0, was used for data analysis. Patient-related and dosimetric data were summarized using descriptive statistics; quantitative variables were expressed as mean ± SD. For each patient, the kV imaging duration of the setup was calculated, and parametric and non-parametric tests (Mann Whitney U) were used to examine differences between groups. The dosimetric data were compared using the two-tailed paired *t* test. The statistical level of significance was set at a *p* value less than 0.05. The statistical analyses were performed using SPSS 21.0.0 (SPSS Inc., Chicago, IL).

## Results

Patient characteristics are presented in Table 1. Compared with non-coached patients, coached patients were older (55.5 versus 46.5, *p* = 0.003) and had a significantly higher BMI (29.95 versus 26.32 kg/m^2^, *p* = 0.006). There were also four patients diagnosed with pulmonary disease among the coached group. Breast cancer T and N stage distribution were similar between the groups (*p* = 0.27, *p* = 0.49). Chemotherapy was administered before radiation therapy in 50% of both groups (*p* = 0.59). The mean radiation prescription doses were similar among the groups: 4479 (± 352) cGy and 4513 (± 359) cGy (*p* = 0.70), respectively. In both groups, most of the patient’s education status was primary school (*p* = 0.65).

**Table 1:** Patient and treatment characteristics

Feature	Coached DIBH	Non-coached DIBH	*p* Value
Patient number	36	28	
Age (median ± SD)	55.5 ± 9.66	46.5 ± 7.79	0.003*
T stage (%)			
Tis I II III	1 (2.8) 23 (63.9) 12 (33.3) 0	2 (7.1) 12 (42.9) 13 (46.4) 1 (3.6)	0.27
N stage			
N0 N1	24 (66.7) 12 (33.3)	17 (60.7) 11 (39.3)	0.49
Education			
Primary school High school University	22 (61.2) 8 (22.2) 6 (16.7)	14 (50) 6 (21.4) 8 (28.6)	0.50
BMI (mean)	29.95	26.32	0.006*
COPD/asthma or heart disease	4	0	0.06
Chemotherapy before radiotherapy	18 (50)	14 (50)	0.59
Dose and fractionation, cGy (mean ± SD)	4479.58 ± 352	4513.75 ± 359	0.70
RT technique(%)			
Conformal IMRT	16 (44) 20 (56)	10 (36) 18 (64)	0.60

*DIBH* deep inspiration breath hold, *COPD* chronic obstructive pulmonary disease, *IMRT* intensity-modulated radiotherapy

^*^p < 0.05

### Set-up duration

**Fig. 2:**
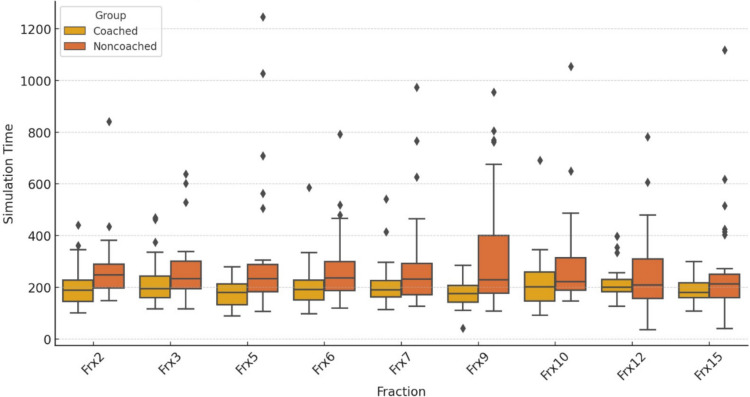
A box-and-whisker diagram showing differences in simulation times of coached and non-coached patients

The set-up preparation time significantly decreased more than half of the fractions in the cDIBH group compared with the ncDIBH group, despite more pulmonary disease, older age, and higher BMI in the coached group. The mean setup times for the cDIBH and ncDIBH were 181.56 s and 280.44 s, respectively. The set-up preparation times of the 16 fractions are listed in Table 2; for the statistically different fractions, a box-and-whisker diagram is shown in Fig. 2 for each group.

**Table 2:** Comparison of treatment set-up duration between cDIBH and ncDIBH patient groups

Treatment fraction	Mean set-up time cDIBH (s) ± SD	Mean set-up time ncDIBH (s) ± SD	*p* value
1^st^ fraction	443 ± 246	530 ± 197	0.134
2^nd^ fraction	187 ± 92	260 ± 139	0.014*
3^rd^ fraction	195 ± 107	268 ± 127	0.04*
4^th^ fraction	189 ± 82	248 ± 192	0.103
5^th^ fraction	156 ± 74	303 ± 278	0.003*
6^th^ fraction	196 ± 99	268 ± 152	0.027*
7^th^ fraction	202 ± 88	287 ± 201	0.027*
8^th^ fraction	195 ± 116	243 ± 105	0.093
9^th^ fraction	166 ± 65	339 ± 240	0.000*
10^th^ fraction	192 ± 125	276 ± 196	0.042*
11^th^ fraction	189 ± 85	239 ± 185	0.157
12^th^ fraction	172 ± 108	253 ± 174	0.026*
13^th^ fraction	193 ± 191	267 ± 223	0.162
14^th^ fraction	181 ± 120	276 ± 355	0.138
15^th^ fraction	168 ± 73	270 ± 209	0.008*
16^th^ fraction	205 ± 104	243 ± 195	0.31

*cDIBH* coached deep inspiration breath hold, *ncDIBH* noncoached deep inspiration breath hold

*p < 0.05

### Cardiac dose and volume exposure

**Fig. 3:**
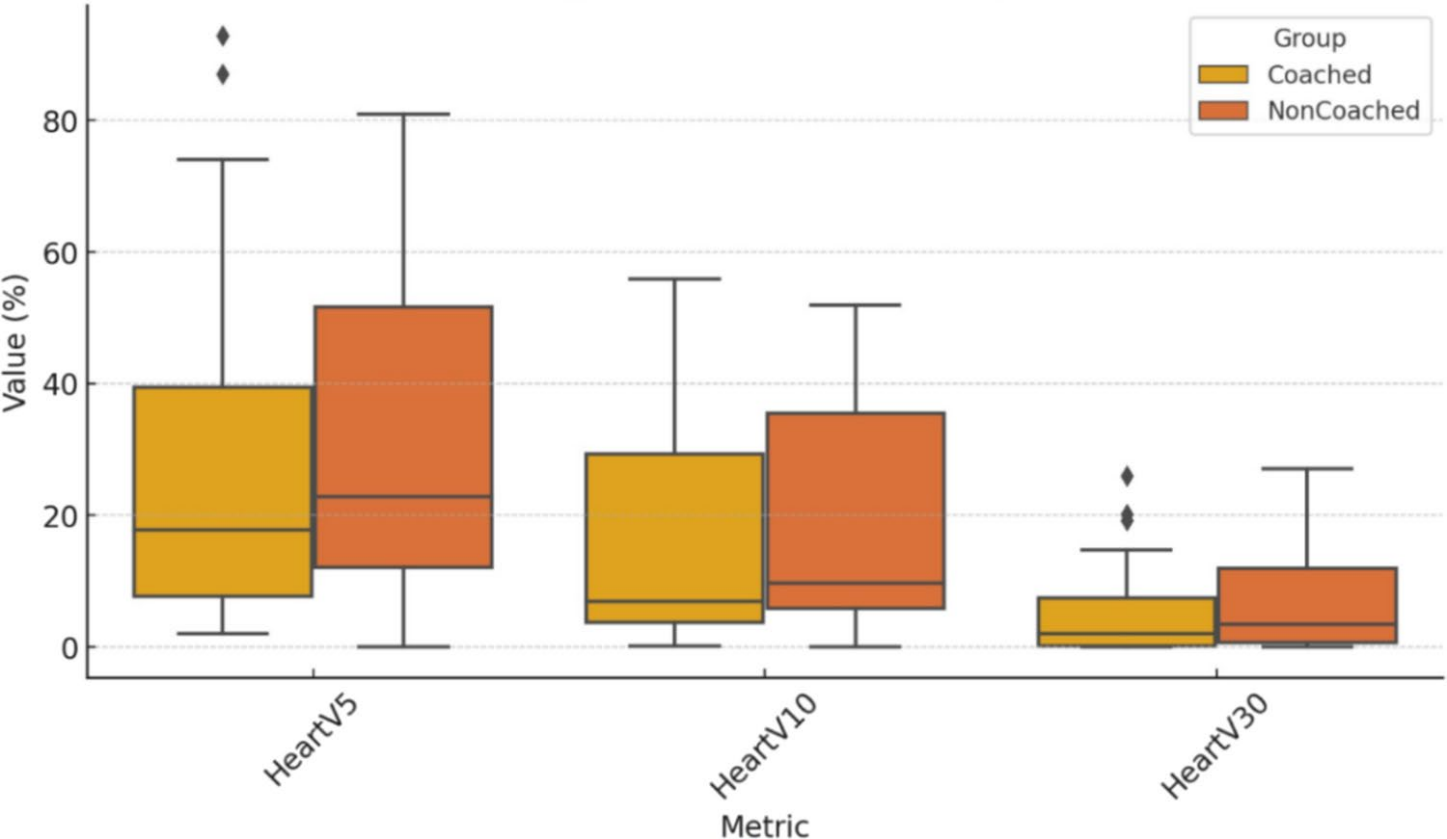
A box-and-whisker diagram showing differences in heart V5, V10, and V30 in percentage

**Fig. 4:**
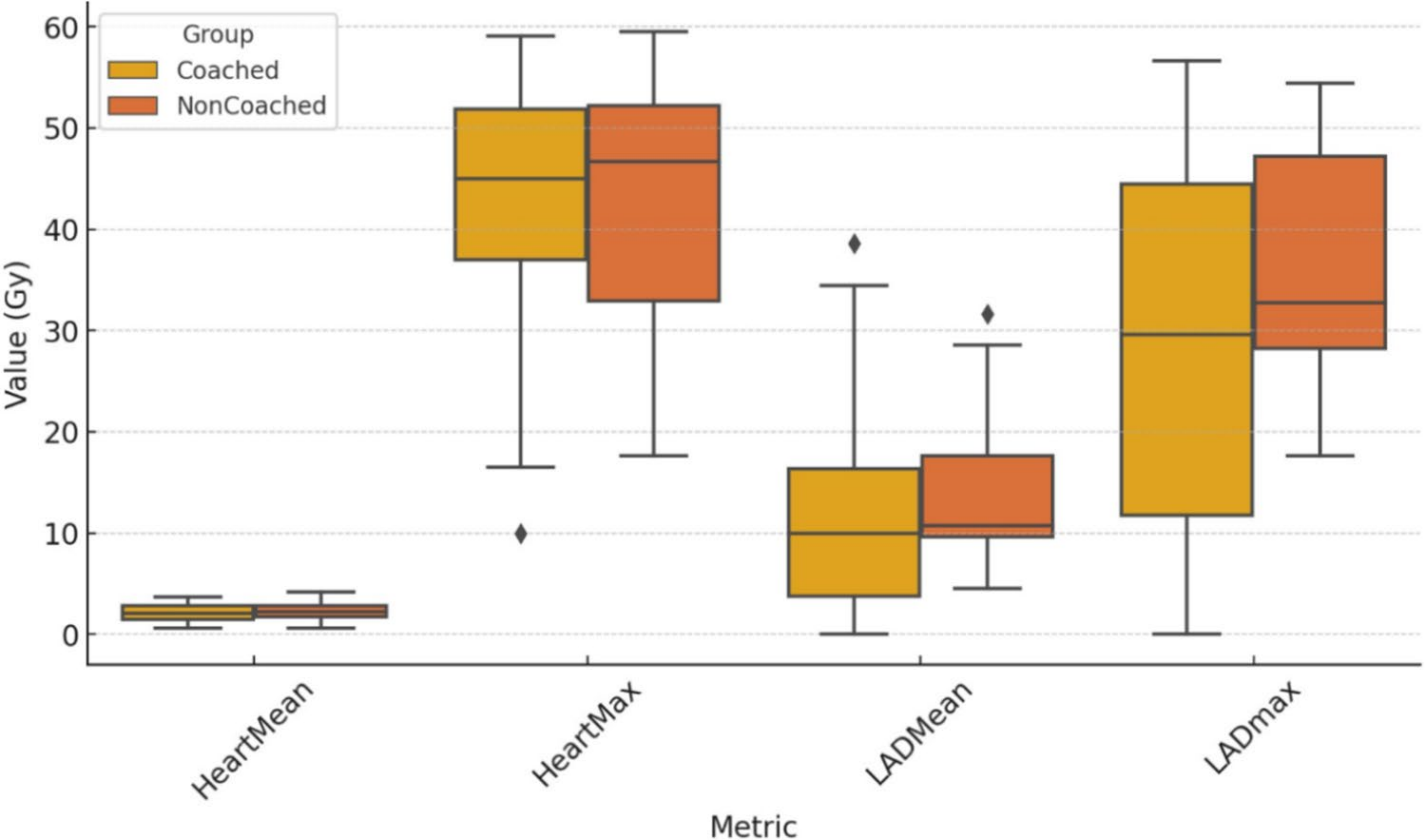
A box-and-whisker diagram showing differences in heart and LAD mean and max doses in Gy

In the coached group, the heart V5 (27.87 versus 31.31, *p* = 0.59), V10 (15.52 versus 17.87, *p* = 0.59), V30 (4.9 versus 6.6, *p* = 0.33), mean (2.06 versus 2.27, *p* = 0.36), and maximum heart doses (42.9 versus 43.58, *p* = 0.82) were lower than those in the non-coached group, but this difference was not statistically significant (Table 3; Fig. 3). LAD max dose was significantly lower in the coached group than in the non-coached group (36.5 versus 29.5, *p* = 0.02, Fig. 4). The mean LAD dose was also lower in the coached group, but the difference was not statistically significant (*p* = 0.38, Fig. 4). Although the left lung volume was higher in the cDIBH (1941 ± 400 cm^3^ vs. 1886 ± 424 cm^3^), the difference was not statistically significant (*p* = 0.59).

**Table 3:** Results of cardiac dosimetric comparison between cDIBH and ncDIBH

	Coached DIBH		Non-coached DIBH		
	Average	± SD	Average	± SD	*P Value*
Heart					
V5	27.87	26.35	31.31	24.52	0.59
V10	15.52	17.46	17.87	17.3	0.59
V30	4.9	6.81	6.6	7.22	0.33
* D*_mean_ (Gy)	2.06	0.97	2.27	0.83	0.36
* D*_max_ (Gy)	42.9	11.45	43.58	12.57	0.82
LAD					
* D*_max_ (Gy)	29.5	17.61	36.5	11.28	0.02*
* D*_mean_ (Gy)	12.04	9.76	13.98	7.24	0.38

*DIBH* deep inspiration breath hold, *LAD* left anterior descending artery

*p < 0.05

## Discussion

Although the advancement of technical methods such as IMRT has improved the preservation rates of normal tissues, different methods are needed to protect the heart and prevent its movement. DIBH was developed as one of the strategies for this purpose [14]. Numerous studies have demonstrated that the DIBH technique provides better cardiac tissue protection compared to free breathing dosimetrically [8, 9, 15]. However, the DIBH technique has disadvantages; It is not standardized, and reports of radiotherapy have shown varied degrees of success. In clinical practice, less is known about standardizing patient preparation for pretreatment simulation. The treatment compliance rates ranged from 73.5 to 87.58% [16–18].

Audio and visual coaching systems were used to increase compliance and visual feedback (VF) system that can control the amplitude is found more appropriate than an audio coaching (AC) system [10]. They found that patients were able to relax more without unnecessary tension and deformation by visually seeing the target of the breath-hold position. This also improved the reproducibility of the chest wall position using respiratory motion and subsequently reduced doses applied to OARs such as the heart and contralateral breast. In our clinic, we do not have a VF system and we treat > 100 patients daily. We have only two lineer accelerators: Varian Trilogy and Siemens Oncor. Since the implementation of DIBH, we have encountered numerous patients who were unable to hold their breath successfully during 4–5 weeks of treatment. DIBH requires a longer simulation time, and patients stay longer in the treatment room, which is a problem for radiotherapy units with a high workload like ours. Patient age and ability to breath-hold for a predetermined length of time were the most common problems for compliance.

In a study focusing on DIBH from the patient’s perspective, participants who received coaching during a practice session at the treatment facility reported feeling relaxed and assured while executing DIBH during their therapy sessions [19]. However, a number of patients suggested doing more self-practice outside of clinical settings. A key component of preparing the respiratory muscles for successful and reliable DIBH during radiation therapy is patient coaching and self-practice with instructions.

This study showed that practicing before the simulation with home practice and instructions significantly reduced the treatment preparation time for most fractions (Table 2; Fig. 2). In the literature, only two studies reported home practice at least 5 days before the CT simulation [11, 12]. In a study by Oonsiri et al., training with an information sheet and educational video at least 1 week before the simulation procedure led to a significant reduction in the simulation time (from 22.3 to 10.3 min) [12]. The addition of an educational video to the instruction sheet did not significantly alter the outcome. The average simulation time decreased dramatically across all age groups and educational levels. In our study, we also did not find any relationship between age and educational status among the groups.

Radiotherapy itself is a stressful and anxiety-provoking medical procedure, which is difficult to manage for most patients. Lewis et al. discovered that anxiety may be highest at the beginning of radiotherapy, especially during the radiotherapy simulation phase and the first radiotherapy session, and that 5–16% of patients presented with clinically relevant anxiety during treatment [20]. This can be attributed to patient anxiety and, more importantly, the coaching required for verifying the treatment setup during the first session (Table 2). Moreover, the ncDIBH group did not improve throughout the fractions (Fig. 2). This highlights the significance of the preparatory phase of the DIBH technique, which was insufficient for the ncDIBH.

In another study that performed home practice before simulation, Kim et al. studied the impact of advance preparatory coaching and home practice. They found that preparatory coaching and training significantly reduced the heart dose [11]. The mean DIBH cardiac dose did not, however, differ significantly between the coached and non-coached groups, as did ours. The reason for this outcome is that the heart’s heterogeneous distribution is not entirely reflected by the mean cardiac dose. The mean heart dose is the most commonly studied dose constraint, but recent data showed that the dose to the LAD artery is a much better predictor of coronary artery disease than MHD alone [21]. They demonstrated that despite MHD < 3 Gy, 56% of patients received LAD doses > 40 Gy. The maximum dose administered to the LAD may have greater significance than the mean dose given that the LAD is a serial structure and that radiation-induced coronary stenosis can occur anywhere along the irradiated mid-to-distal segment. Our study did not find a statistically significant difference in MHD between the groups, but it showed that coaching decreased the max LAD dose further with DIBH (29.5 Gy versus 36.5 Gy) (Table 3).

We observed a difference in lung volumes between coached and non-coached patients. The lung volumes of coached patients were higher (1941 ± 400 cm^3^) compared to non-coached patients (1886 ± 424 cm^3^). However, this difference was not statistically significant, likely due to the insufficient sample size. The differences in heart doses are not solely related to lung volumes; some thoracic anatomical parameters are also discussed in the literature [22]. Although these parameters are outside the scope of our study, they warrant further investigation. Despite the lack of standard clinical implementation, the DIBH technique is increasingly used to treat breast and other anatomical regions, such as the lung and abdomen. Motion management training is essential for completing radiation therapy. To emphasize this issue, ESTRO published a guideline that highlights patient compliance and reviews considerations for equipment, staff training, and patient coaching [23]. In this guideline, patient education using materials (flyers, videos, or slide presentations) is recommended to use at home for breath hold and patient training before the CT is advised.

### Limitations

The most important limitations were the retrospective design and the small number of patients. The groups were equally distributed except for a higher BMI (29.95 versus 26.32 kg/m^2^, *p* = 0.006) and higher incidence of pulmonary disease in the coached group, which may have favored the non-coached group. Additionally, we do not have free-breathing CT data for our patients to compare with DIBH plans because not all patients had a dosimetric benefit from using DIBH. Another limitation is the lack of intrafractional accuracy, as whether home practicing improves patient positioning in kV images, which will be an important topic for subsequent future investigations.

However, given the scarcity of concrete clinical data and the small number of studies looking into patient coaching for home practice, this study is valuable for the literature.

## Conclusion

The DIBH technique requires patient training and education. Patient coaching with an educated nurse and home instruction material before the simulation decreased our workload on the linac. The LAD maximum dose is also reduced using this method, representing the primary long-term challenge of left-breast radiation. To demonstrate greater benefits, prospective studies with a large number of patients should be conducted.

## Data Availability

No datasets were generated or analysed during the current study.
